# Influence of grape seeds on wine composition and astringency of Tempranillo, Garnacha, Merlot and Cabernet Sauvignon wines

**DOI:** 10.1002/fsn3.1627

**Published:** 2020-06-02

**Authors:** Jordi Gombau, Pere Pons‐Mercadé, Marta Conde, Lucie Asbiro, Olga Pascual, Sergio Gómez‐Alonso, Esteban García‐Romero, Joan Miquel Canals, Isidro Hermosín‐Gutiérrez, Fernando Zamora

**Affiliations:** ^1^ Facultat d’Enologia de Tarragona Departament de Bioquímica i Biotecnologia Universitat Rovira i Virgili Tarragona Spain; ^2^ Instituto Regional de Investigación Científica Aplicada Universidad de Castilla‐La Mancha Ciudad Real Spain; ^3^ Instituto de la Vid y el Vino de Castilla‐La Mancha Ciudad Real Spain

**Keywords:** astringency, berry morphology, proanthocyanidins, seeds

## Abstract

**Background:**

The aim of this work was to study the influence of grape berry morphology, especially the seed weight percentage, on the tannin concentration and astringency of red wine. Clusters of Tempranillo, Garnacha, Merlot, and Cabernet Sauvignon were characterized and their seeds were extracted and macerated in a model wine solution. In parallel, we elaborated three types of wines of each cultivar. One wine was made with only grape juice, one wine was made adding the appropriate proportion of seeds to the grape juice, and the last wine was elaborated with the complete destemmed and crushed berries.

**Results:**

Merlot and Cabernet Sauvignon grapes, which have higher percentage of seed weight with respect to the berry weight than Tempranillo and Garnacha grapes originated wines with higher tannin concentration and astringency than Tempranillo and Garnacha wines.

**Conclusion:**

The main conclusion of this study is that the seed weight percentage with respect to the berry weight is one of the main determinants of the final tannin concentration and astringency of red wines.

## Graphical abstract

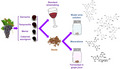

## 1 INTRODUCTION

Phenolic compounds are a large family of molecules that are mainly present in the solid parts of grapes (seed, skin, and stems). These compounds are generally considered to be major determinants of the quality of red wines. Most of the main sensory attributes of wine, such as color, body, mouthfeel, bitterness, and astringency are associated with their phenolic compound composition (Vidal et al., 2004).

The color of red wine is mainly due to anthocyanins (He et al., 2012) and is strongly conditioned by pH, the presence of sulfur dioxide and copigmentation phenomena. Specifically, phenolic acids, flavonols, and flavanols, among other compounds, can greatly influence wine color because they can act as copigments (Boulton, 2001). However, anthocyanins in turn can react with other molecules, especially flavanols (flavan‐3‐ol monomers and proanthocyanidins), to produce new and more stable pigments (Francia‐Aricha, Guerra, Rivas‐Gonzalo, & Santos‐Buelga, 1997; He et al., 2012). In additional, cycloaddition reactions between anthocyanins and other small molecules can produce a new family of anthocyanin‐derived pigments called pyranoanthocyanins (Bakker & Timberlake, 1997; Cheynier et al., 2006).

Flavanols (which include flavan‐3‐ol monomers and proanthocyanidins) are the main determinants of the astringency and bitterness perception in red wine (Peleg, Gacon, Schlich, & Noble, 1999; Vidal et al., 2003). It has been described that the higher the mean degree of polymerization (mDP) and higher the proportion of (‐)‐epicatechin‐3‐*O*‐gallate of the proanthocyanidins the greater the astringency perception (Sun et al., 2013; Vidal et al., 2003). It is generally accepted that seed proanthocyanidins are more astringent than skin proanthocyanidins because they have a higher proportion of epicatechin‐3‐*O*‐gallate. In contrast, flavanol‐3‐ols monomers and proanthocyanidins with lower mDP seem to enhance bitterness perception (Peleg et al., 1999).

Anthocyanins are released from grape skins whereas proanthocyanidins, also called condensed tannins, are released from skins and seeds during the fermentation/maceration process. However, the chemical composition of the proanthocyanidins from seeds and skins is not identical. Grape seed proanthocyanidins are polymers composed of (+)‐catechin, (−)‐epicatechin and (−)‐epicatechin‐3‐*O*‐gallate (Prieur, Rigaud, Cheynier, & Moutounet, 1994). Grape Skin proanthocyanidins are composed of the same monomers but also contain (−)‐epigallocatechin, and the proportion of (−)‐epicatechin‐3‐*O‐*gallate is much lower (Souquet, Cheynier, Brossaud, & Moutounet, 1996). Consequently, grape skin tannins are composed of procyanidins and prodelphinidins because their acidic cleavage gives cyanidin and delphinidin, whereas grape seed tannins are composed only of procyanidins. In addition, seed proanthocyanidins have a lower degree of polymerization (mDP) than skin proanthocyanidins (Prieur et al., 1994). Consequently, grape skins release procyanidins and prodelphinidins with a higher mDP, whereas grape seeds only release procyanidins with a higher proportion of galloylation and a lower mDP.

The composition of phenolic compounds and consequently the quality of red wine depends on several factors, including, the cultivar, (Ortega‐Regules et al., 2008) grape maturity, (Gil et al., 2012) ethanol content, (Canals, Llaudy, Valls, Canals, & Zamora, 2005) fermentation temperature, (Pérez‐Navarro, García‐Romero, Gómez‐Alonso, & Izquierdo‐Cañas, 2018) maceration length (Gil et al., 2012) and the winemaking techniques applied (Canals, del Carmen, Canals, & Zamora, 2008; Lee, Kennedy, Devlin, Redhead, & Rennaker, 2008; Pascual et al., 2016). However, the grape variety used to obtain the wine is probably one of the main factors affecting the composition of phenolic compounds in the wine. In that sense, the morphology of bunches and grapes, which depends largely on the grape cultivar, should play a very important role in the final wine composition. The weight of the bunches, the proportion of the grape weight regardless of the bunch weight, the berry size (weight and volume), and the proportion of seeds weight regardless of the berry weight, have a great influence on the final red wine composition. All these parameters are mainly conditioned by the grape cultivar. Other external factors such as the composition and fertility of the soils, the height of the vineyard, the climatic conditions of the vintage (water availability, sunlight exposure, and temperature), and viticulture practices can also influence these parameters (Chacón, García, Martínez, Romero, & Gómez, 2009; Dokoozlian & Kliewer, 1996; Holt, Francis, Field, Herderich, & Iland, 2008) but to a lesser extent than the genotypic characteristics.

In a relatively recent article, Gil et al., (2015) reported that the berry size greatly influences the color and composition of phenolic compounds of Cabernet Sauvignon wines elaborated with grapes from the same vineyard. Specifically, the smaller the berry size the more intense the color and the higher the concentration of anthocyanins and proanthocyanidins. Moreover, Gil et al. (2015) reported that the wines obtained from small berries have a higher proportion of prodelphinidins and lower proportion of galloylated subunits than wines obtained with larger berries. These data suggest that small berries have a higher proportion of skin proanthocyanidins and a lower proportion of seed proanthocyanidins than large berries because prodelphinidins are only present in skins, and seed proanthocyanidins are richer in galloylated subunits. In fact, these data can be considered as very logical since the smaller berries have a higher skin‐to‐flesh ratio (Bindon, Myburgh, Oberholster, Roux, & Du Toit, 2011) and usually also have a lower number of seeds (Barballado, Guidoni, & Hunter, 2011; Shellie, 2010). However, the skin‐to‐flesh ratio can be also conditioned by the berry skin thickness. Recently, Gil, Úbeda, del Barrio‐Galán, and Peña‐Neira (2020) have reported that berries of higher size have lower surface‐to‐volume ratio but higher skin thickness than berries of lower size. Gil et al. (2020) also reported that larger berries have higher proportion of skins, mainly due to their higher skin thickness.

It is generally considered that not well‐ripened grapes may produce more astringent and bitter wines because their seeds can release a larger amount of proanthocyanidins, which are highly galloylated (Prieur et al., 1994). Therefore, winemakers usually consider the phenolic maturity of the grapes, and especially of the grape seeds, as a major parameter for deciding the harvest date. Some approaches have even been proposed to eliminate seeds during the winemaking process when the grape seeds are not well lignified (Canals et al., 2008; Lee et al., 2008).

The objective of this research was to study the relationship between the morphology of bunches and grapes with similar maturity levels, especially the seed‐to‐flesh ratio, of four different red *Vitis vinifera* cultivars (Tempranillo, Garnacha, Merlot, and Cabernet Sauvignon) from different vineyards and the final composition and astringency of the wines obtained with these grapes. The grape morphology depends largely on the grape variety; however, it is evident that the genetic characteristics of each cultivar, the different edaphoclimatic conditions of the vineyard, and the viticulture practices can condition the phenolic composition of wines elaborated with grapes of each one of these cultivars (Chacón et al., 2009; Dokoozlian & Kliewer, 1996; Holt et al., 2008).

## 2 MATERIALS AND METHODS

### 2.1 Chemicals

Methanol, formic acid, and acetic acid were of HPLC grade and were purchased from Panreac (Barcelona, Spain). Acetaldehyde, phloroglucinol, sodium acetate, ammonium acetate, sodium hydroxide, tannic acid, methylcellulose, albumin from chicken egg, ascorbic acid, and ammonium formate were purchased from Sigma‐Aldrich. Absolute ethanol and hydrochloric acid were purchased from Panreac. Malvidin‐3‐*O*‐glucoside chloride (95%) and (‐)‐epicatechin (99%) were purchased from Extrasynthese.

### 2.2 Grapes and wines

The experiment was carried out with *Vitis vinifera* cv. grapes of Tempranillo Tinto (Variety Number VIVC: 12350 (Vitis International Variety Catalogue) and Garnacha Tinta (Variety Number VIVC: 4461 (Vitis International Variety Catalogue) from the Cal Bessó estates at *Els Guiamets* (AOC Montsant, Tarragona, Spain; 41°6′15.2893″ (N) and 0°45′47.9652″ (E); at a height of 230 m above sea level) and Merlot Noir (Variety Number VIVC: 7657 (Vitis International Variety Catalogue,)) and Cabernet Sauvignon (Variety Number VIVC: 1929 (Vitis International Variety Catalogue) from the Juvé & Camps estates at *Mediona* (AOC Penedès, Barcelona, Spain; 41°31′30.1080″ (N) and 1°42′47.4516″ (E); at a height of 590 m above sea level) during of the 2016 vintage. The grapes were harvested manually when they had reached the appropriate maturity to obtain high quality red wines (between 23.5 and 24.5°Brix). One hundred kg of grapes of each cultivar were harvested.

### 2.3 Morphologic characterization of the grape clusters

Ten clusters were used for the morphologic characterization. Specifically, the following parameters were determined: cluster weight (CW), stem weight of a cluster (SCW), the weight of all the berries in a cluster (BCW), the percentage of stem weight with respect to cluster weight (%SC), the percentage of berry weight with respect to cluster weight (%BC) and the number of berries per cluster (NBC). The weight (100BW) and volume (100BV) of 100 berries were also measured in triplicate. The seeds of 100 berries were then extracted and used for determining in triplicate the number of seeds per berry (NSB) and the weight of 100 seeds (100SW). These data were used to calculate the percentage of seed weight with respect to berry weight (%SB).

### 2.4 Microvinifications

**FIGURE 1:**
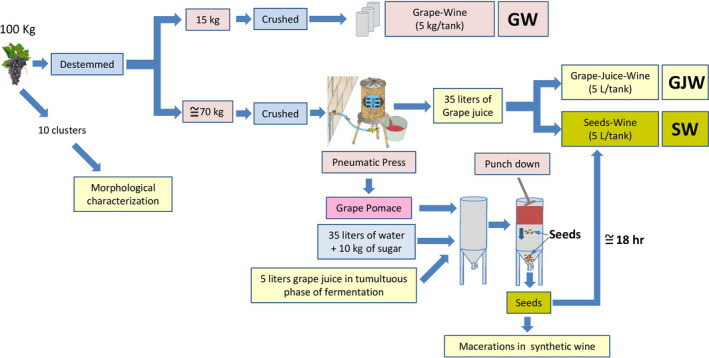
Experimental design

Figure 1 shows a schematic diagram of the experimental design. Hundred kg of clusters of each variety were carefully destemmed (Delta V2, Bucher Vaslin SA) without affecting the integrity of the berries. First, 15 kg of destemmed berries were grouped into three batches of 5 kg of berries that were immediately crushed, sulfited (100 mg of K_2_S_2_O_5_/Kg), and placed in three fermentation tanks for conventional microvinifications in triplicate (Grape Wine; GW). Second, 70 kg of destemmed grapes were crushed and immediately pressed in a pneumatic press. Around 35 L of grape juice were extracted and sulfited (100 mg of K_2_S_2_O_5_/Kg). Six batches of 5 L of this grape juice were poured into six fermentation tanks. Three of them were fermented to obtain a wine originated only from the grape juice without the presence of any other part of the cluster (Grape Juice–Wine; GJW). The other three batches were supplemented with the adequate proportion of seeds (%SB) that had been determined previously (Seed Wine; SW).

All tanks were immediately inoculated with 200 mg/kg selected yeast (EC1118, Lallemand Inc.). All tanks were kept at 27 ± 1°C. After 15 days of maceration, the wines from the tanks were racked. All wines were sulfited (100 mg K_2_S_2_O_5_/L) and kept at 4°C for 1 month for tartaric stabilization. Malolactic fermentation was therefore inhibited so as to prevent it from causing any variations. The wines were finally bottled and stored in a dark cellar at 15°C until analysis. The wines were analyzed between 3 and 6 months after bottling.

### 2.5 Seeds isolation

The remaining pomace obtained in the pressing process was placed in a conical bottom tank with an extraction tap just at the apex of the cone. This tank was immediately supplemented with 35 L of water, 10 kg of commercial sucrose, and 5 L of white grape juice in the tumultuous fermentation step to induce the rapid beginning of alcoholic fermentation. A few hours later, when the cap was already formed, several manual punch downs were vigorously performed so the seeds would fall to the bottom of the tank. This process was repeated five times during the day. The following morning, around 18 hr later, the extraction tap was opened and the seeds were taken out using a colander. This was done as quickly as possible to minimize the extraction of phenolic compounds from the seeds. The seeds were then washed in cold water (4°C), and the small proportion of skins was manually separated. The seeds were then dried using a hair dryer until their weight was stable.

### 2.6 Seeds maceration

A volume of 240 ml of a model wine solution (ethanol 13.5% v/v, tartaric acid 4.0 g/L adjusted at pH 3.50 with sodium hydroxide) was used to macerate the seeds of each cultivar. The seed weight was calculated using the percentage of seed weight with respect to the berry weight (%SB) considering that the theoretical volume of the grape juice was 80% of the grape berry weight for all cultivars. Macerations were performed in triplicate in closed dark flasks of 250 ml at 27°C. After 15 days of maceration, the seeds were separated and the wine model solution was centrifuged (5 min at 12,000 x *g*). The macerated solutions were stocked at 4°C until analysis.

### 2.7 Standard grape juice analysis

The analytical methods recommended by the International Organization of Vine and Wine (Organisation Internationale de la Vigne et du Vin, 2014) were used to determine the total soluble solids expressed as °Brix, the potential ethanol content (% v/v), titratable acidity and pH of the grape juice. These measurements were taken in triplicate using the grape berries obtained by manually destemming the clusters used for morphological characterization of the clusters.

### 2.8 Standard wine analysis

The ethanol content, titratable acidity, and pH of wines were determined by the methods recommended by OIV (Organisation Internationale de la Vigne et du Vin, 2014). The total polyphenol index (TPI) was determined by measuring the 280 nm absorbance of a 1:100 wine dilution, with a spectrophotometer, using a 10 mm quartz cuvette according to Ribéreau‐Gayon, Glories, Maujean and Dubourdieu (2006) The total anthocyanin content was determined by spectrophotometry using the method described by Niketic‐Aleksic and Hrazdina (1972) The total tannin content was determined by methylcellulose method described by Sarneckis et al. (2006).

### 2.9 Color parameters

The color intensity (CI) was determined by the method described by Glories (1984) The CIEL*a*b* coordinates (Lighness; L*, Chroma; C* Hue; H*) were determined by the method described by Ayala, Echavarri and Negueruela (1997), and data processing was performed with MSCV software (Ayala, Echávarri, & Negueruela, 2013).

### 2.10 Anthocyanin analysis by HPLC

Reversed‐phase HPLC analyses of the anthocyandins were carried out by injecting 40 µl of wine into an Agilent 1200 series liquid chromatograph (HPLC‐DAD) and using an Agilent Zorbax Eclipse XDBC18, 4.6 × 250 mm, 5 µm column (Agilent Technologies). The solvents used were 10% aqueous formic acid (solvent A) and a mixture of 45% methanol, 45% water, and 10% formic acid (solvent B) in accordance with the method described by Gil et al. (2015) Chromatograms were recorded at 530 nm, and anthocyanin standard curves were made using malvidin‐3‐*O*‐glucoside chloride.

### 2.11 Analysis of proanthocyanidins by phloroglucinolysis

Acid‐catalyzed depolymerization of proanthocyanidins in the presence of an excess of phloroglucinol was used to analyze the content of proanthocyanidins, their monomeric composition, and their mean degree of polymerization (mDP), as described by Kennedy and Jones (2001) A 10 ml sample of wine was evaporated under a low‐pressure vacuum (Univapo 100 ECH, Uni Equip). It was subsequently resuspended in 6 ml of distilled water and then applied to Set Pak Plus tC18 Environmental cartridges (Waters) that had previously been activated with 10 ml of methanol and 15 ml of water. The samples were washed with 15 ml of distilled water. The proanthocyanidins were then eluted with 12 ml of methanol, immediately evaporated under a vacuum, and re‐dissolved in 2 ml of methanol. Finally, 100 µl of this sample was reacted with a 100 µl phloroglucinol solution (0.2 N HCl in methanol, containing 100 g/L phloroglucinol and 20 g/L ascorbic acid) at 50°C for 20 min. The reaction was stopped by adding 1,000 ml of 40 mM aqueous sodium acetate. Reversed‐phase HPLC analysis (Agilent series 1200 HPLC‐DAD) was carried out with an Agilent Zorbax Eclipse XDBC18, 4.6 × 250 mm, 5 µm column (Agilent Technologies) as described below, and the injection volume was 30 µl. The solvents used were 1% aqueous acetic acid (solvent A) and methanol (solvent B) at a flow rate of 1 ml/min. The elution conditions were 1.0 ml/min. Elution was performed with a gradient starting at 5% B for 10 min, a linear gradient from 5% to 20% B in 20 min, and a linear gradient from 20% to 40% B in 25 min. The column was then washed with 90% B for 10 min and re‐equilibrated with 5% B for 5 min before the next injection. The monomers (+)‐catechin, (‐)‐epicatechin, and (‐)‐epicatechin‐3‐*O*‐gallate were identified by comparing their retention times with those of the pure compounds. The phloroglucinol adducts of (+)‐catechin, (‐)‐epicatechin, (‐)‐epigallocatechin, and (‐)‐epicatechin‐3‐*O*‐gallate were identified by their retention time and confirmed through an HPLC‐MS analysis. Analyses were performed with Agilent 1200 series HPLC using an Agilent 6210 time‐of‐flight (TOF) mass spectrometer equipped with an electrospray ionization system (ESI). Elution was without adding acid and phloroglucinol carried out under the same HPLC analysis conditions described below. The capillary voltage was 3.5 kV. Nitrogen was used both as a dry gas at a flow rate of 12 L/min at 350°C and as a nebulizer gas at 60 psi. Spectra were recorded in positive ion mode between m/z 50 and 2,400.

This assay was also carried out without adding acid and phloroglucinol to measure the flavan‐3‐ol monomers that are naturally present in wine. The number of terminal subunits was considered to be the difference between the total monomers measured in normal conditions (with phoroglucinol and acid) and the monomers measured when the analysis was performed without adding acid and phloroglucinol. The number of extension subunits was considered as the addition of all the phloroglucinol adducts. The mDP was calculated by adding the terminal and extension subunits (in moles) and dividing by the terminal subunits. The percentage of galloylation (%GAL) was calculated considering the molar percentage of the monomer (‐)‐epicatechin‐3‐*O*‐gallate. The percentage of prodelphinidins (%PD) was calculated considering the molar percentage of the monomer (‐)‐epigallocatechin. This last parameter was only measured in the GW since prodelphinidins are only present in the skins (Souquet et al., 1996).

### 2.12 Astringency index

The astringency of the wines and the model wine solutions were determined by the Astringency Index method described by Llaudy et al. (2004).

### 2.13 Statistical analysis

All of the chemical and physical data for the samples are expressed as the arithmetic average ± standard deviation of three replicates. One‐factor univariate analysis of variance (ANOVA) was carried out with the SPSS software (SPSS Inc.) in order to compare between cultivars The statistical differences were established at *p* < .05. Principal component analysis (PCA), using a XLSTAT software, were performed in order to better understand which factors are the main determinants of the astringency of the grape wines (GW).

## 3 RESULTS AND DISCUSSION

Table 1 shows the morphologic characterization of the clusters and berries of each variety. The weights of the Cabernet Sauvignon and Merlot clusters (CW) were significantly lower than those of the Garnacha and Tempranillo clusters. However, in the Tempranillo and Garnacha varieties around 4% of the cluster weight was due to stems (%SC), whereas for Cabernet Sauvignon and Merlot stem weight was around 7%. The percentage of berry weight with respect to cluster weight (%BC) was only slightly, but significantly, higher in Garnacha and Tempranillo than in Merlot and Cabernet Sauvignon. Moreover, the number of berries per cluster (NBC), the weight of all the berries in a cluster (BCW), and the weight (100BW) and the volume (100BV) of 100 berries were the higher for the Tempranillo and Garnacha than for Merlot and Cabernet Sauvignon.

**TABLE 1:** Morphologic characterization of the grape clusters

Parameter	Garnacha	Tempranillo	Merlot	Cabernet Sauvignon
CW (g)	298.8 ± 89.5 B	350.1 ± 92.6 B	121.3 ± 39.2 A	128.6 ± 44.0 A
SCW (g)	11.53 ± 2.49 A	12.50 ± 3.92 A	8.14 ± 2.57 A	9.54 ± 3.99 A
BCW (g)	286.7 ± 3.4 C	337.4 ± 0.3 D	113.1 ± 3.0 A	119.1 ± 0.5 B
%BC	95.97 ± 0.85 B	96.36 ± 0.22 B	93.19 ± 1.23 A	92.58 ± 1.89 A
%SC	4.03 ± 0.85 A	3.64 ± 0.31 A	6.81 ± 1.09 B	7.42 ± 0.75 B
NBC	173.0 ± 18.7 C	177.5 ± 5.1 C	99.4 ± 16.1 A	122.9 ± 8.3 B
100BW (g)	165.7 ± 17.9 B	190.1 ± 5.4 C	113.8 ± 18.4 A	96.9 ± 6.6 A
100BV (ml)	151.2 ± 16.2 B	172.8 ± 5.2 B	103.4 ± 16.3 A	88.3 ± 6.1 A
NSB	1.37 ± 0.12 A	1.71 ± 0.12 B	2.07 ± 0.21 B	1.83 ± 0.10 B
100SW (g)	2.44 ± 0.07 A	3.48 ± 0.41 B	3.58 ± 0.43 B	3.08 ± 0.49 B
%SB	2.03 ± 0.13 A	3.14 ± 0.40 B	6.53 ± 1.43 C	5.82 ± 0.62 C

Note

All data are expressed as the average values ± standard deviation. Different letters indicate the existence of statistical differences between different cultivars (*p* < .05).

Abbreviations: %BC, percentage of berry weight with respect to cluster weight; %SB, percentage of seed weight with respect to berry weight; %SC, percentage of stem weight with respect to cluster weight; 100BV, volume of 100 berries; 100BW, weight of 100 berries; 100SW, weight of 100 seeds; BCW, weight of all the berries in a cluster; CW, cluster weight; NBC, number of berries per cluster; NSB, number of seeds per berry; SCW, stem weight of a cluster.

Table 1 also shows the number of seeds per berry (NSB), the weight of 100 seeds (100SW) and the percentage of seed weight with respect to berry weight (%SB) for each variety. The number of seeds per berry (NSB) and the weight of 100 seeds (100SW) were significantly lower in Garnacha than in the other three cultivars, which had similar values. Therefore, the percentage of seed weight with respect to berry weight (%SB) was the lowest in Garnacha followed in increasing order by Tempranillo, Cabernet Sauvignon and Merlot, although the difference between these two last cultivars was not statistically significant.

It has been reported that the berry size greatly influences the phenolic composition of Cabernet Sauvignon wines (Gil et al., 2015). In brief, the smaller the berry size the higher the anthocyanin and proanthocyanidin concentrations. These data can probably be extrapolated to other cultivars. If this hypothesis is true it would be expected that wines of Garnacha and Tempranillo would have a lower concentration of anthocyanins and proanthocyanidins than wines of Merlot and Cabernet Sauvignon since their berries are significantly bigger than those of these last two cultivars. However, the grape skin thickness of the berries of each cultivar can also determine the skin proportion during the winemaking process and consequently the amount of phenolic compounds released from skins (Gil et al., 2020). Moreover, other factors such as the genetic characteristics (Ortega‐Regules et al., 2008), the grape maturity (Gil et al., 2012), the edaphoclimatic conditions of the vineyards and viticulture practices (Chacón et al., 2009; Dokoozlian & Kliewer, 1996; Holt et al., 2008) also have a major influence on the final amount of anthocyanins and proanthocyanidins and, also on their extractability. In addition, the proportion of seeds with respect to grape juice differs widely between varieties, which may also be determinant of the amount of phenolic compounds released by seeds. Consequently, this could have a nonnegligible effect on the color and the final chemical composition of red wine elaborated with each of these varieties.

Table 2 shows the grape juice composition of the wines. The four cultivars were harvested with a total soluble solid content between 23.5 and 24.5°Brix, which represent a potential ethanol content between 13.6% and 14.4% (v/v). The titratable acidity of the four varieties was between 5.15 and 5.96 g/L (expressed as tartaric acid) and the pH was between 3.04 and 3.35. In general, these parameters indicate that the maturity level of the different grape cultivars was similar and adequate for the elaboration of premium red wines, although some small but sometimes significant differences were found.

**TABLE 2:** Grape juice composition of the four cultivars

Parameter	Garnacha	Tempranillo	Merlot	Cabernet Sauvignon
Soluble solids (°Brix)	23.8 ± 0.2 A	23.5 ± 0.1 A	24.5 ± 0.2 B	24.5 ± 0.2 B
Potential ethanol content (% v/v)	13.8 ± 0.1 A	13.6 ± 0.2 A	14.3 ± 0.2 B	14.4 ± 0.1 B
Titratable acidity (g/L)	5.87 ± 0.23 B	5.96 ± 0.12 B	5.37 ± 0.17 A	5.15 ± 0.21 A
pH	3.04 ± 0.02 A	3.10 ± 0.03 B	3.14 ± 0.02 B	3.35 ± 0.02 C

Note

All data are expressed as the average values of three replicates ± standard deviation. Different letters indicate the existence of statistical differences between different cultivars (*p* < .05).

Table 3 shows the general parameters of the different wines obtained from the four varieties. As explained above, three different wines were obtained for each variety depending on whether the whole crushed grapes were macerated and fermented (Grape wine; GW), only the grape juice was fermented (Grape juice wine; GJW) or the grape juice was macerated and fermented with the appropriate proportion of seeds (Seed wine; SW). The ethanol content of the different wines was between 13.7% and 14.0% (v/v) in the case of Garnacha, between 13.6% and 13.9% in the case of Tempranillo, between 14.5% and 14.7% in the case of Merlot, and between 14.4% and 14.7% in the case of Cabernet Sauvignon. In all the cases these ethanol content levels were similar between the different winemaking types for each variety and also very similar to the potential ethanol content of the corresponding grapes (Table 2). Titratable acidity was between 6.05 and 6.30 g/L for Garnacha, between 5.88 and 6.03 g/L for Tempranillo, between 5.40 and 5.63 g/L for Merlot, and between 5.25 and 5.30 g/L for Cabernet Sauvignon. Once again, these values are similar to values obtained in their corresponding grapes. However, titratable acidity seems to be significantly lower when the skins are present in the fermentation media for Garnacha and Tempranillo. The pH values were between 3.04 and 3.15 for Garnacha, between 3.09 and 3.41 for Tempranillo, between 3.18 and 3.37 for Merlot, and between 3.45 and 3.76 for Cabernet Sauvignon. The pH values of GJW and SW wines were similar to those of their corresponding grapes (Table 2). However, the pH of GW wine was significantly higher than in GJW and SW wines in all the varieties. The decrease in the titratable acidity of Garnacha and Tempranillo wines and the increase in pH of all cultivars observed in the GW wines is probably related to the release of potassium from the skins (Harbertson & Harwood, 2009). Potassium can react with tartaric acid to form potassium hydrogen tartrate which can precipitate and therefore cause increases in pH.

**TABLE 3:** Wine general parameters

Parameter	Garnacha	Tempranillo	Merlot	Cabernet sauvignon
Ethanol content (%)				
GW	14.0 ± 0.2 Aα	13.9 ± 0.1 Aα	14.5 ± 0.1 Bα	14.5 ± 0.1 Bα
GJW	13.8 ± 0.1 Aα	13.6 ± 0.1 Aα	14.7 ± 0.1 Bα	14.7 ± 0.1 Bα
SW	13.7 ± 0.1 Aα	13.7 ± 0.1 Aα	14.5 ± 0.1 Bα	14.4 ± 0.2 Bα
Titratable acidity (g/L)				
GW	6.05 ± 0.03 Dα	5.88 ± 0.04 Cα	5.63 ± 0.07 Bα	5.25 ± 0.05 Aα
GJW	6.25 ± 0.05 Dβ	6.00 ± 0.05 Cβ	5.40 ± 0.10 Bα	5.25 ± 0.05 Aα
SW	6.30 ± 0.05 Cβ	6.03 ± 0.06 Bβ	5.50 ± 0.15 Aα	5.30 ± 0.06 Aα
pH				
GW	3.15 ± 0.01 Aβ	3.41 ± 0.01 Cβ	3.37 ± 0.01 Bβ	3.76 ± 0.02 Dβ
GJW	3.05 ± 0.01 Aα	3.11 ± 0.02 Bα	3.16 ± 0.03 Bα	3.42 ± 0.03 Cα
SW	3.04 ± 0.02 Aα	3.09 ± 0.02 Aα	3.18 ± 0.01 Bα	3.45 ± 0.01 Cα

Note

All data are expressed as the average values of 3 replicates ± *SD*. Different capital letters indicate the existence of statistical differences between cultivars (*p* < .05) and different Greek letters indicate the existence of statistical differences between wines of the same cultivar (*p* < .05).

Abbreviations: GJW, Grape juice wine; GW, Grape wine; SW, Seeds wine.

[Correction added on 22 June 2020, after first online publication: the content of Table 3 has been replaced.]

Table 4 shows the phenolic compounds of the model wine solutions obtained by maceration of the seeds of each variety. Evidently, these maceration conditions do not reproduce exactly what happens during alcoholic fermentation where ethanol is produced progressively; however, it provides an approximate idea of the releasing capacity of the seeds of each variety under the same media conditions.

**TABLE 4:** Total polyphenol index, tannin concentration, proanthocyanidin concentration, and astringency index of the different seed maceration samples

Parameter	Garnacha	Tempranillo	Merlot	Cabernet Sauvignon
IPT	18.3 ± 0.7 A	16.9 ± 0.8 A	69.4 ± 3.8 C	54.2 ± 0.9 B
Tannins				
g/L	0.93 ± 0.10 A	1.00 ± 0.16 A	4.68 ± 0.32 C	3.54 ± 0.24 B
mg/g of seeds	45.8 ± 4.9 B	31.8 ± 5.0 A	71.7 ± 5.0 D	60.8 ± 4.1 C
Proanthocyanidins				
Concentration (mg/L)	464 ± 28 A	451 ± 55 A	1503 ± 82 C	1,144 ± 120 B
mDP	3.9 ± 0.3 A	4.0 ± 0.2 A	4.2 ± 0.5 A	3.9 ± 0.3 A
% Galloylation	18.2 ± 0.6 B	19.9 ± 0.2 C	19.4 ± 0.3 C	16.8 ± 0.4 A
Astringency index				
mg Tannic ac./L	158 ± 49 A	330 ± 40 B	1,031 ± 50 D	640 ± 32 C
mg Tannic ac./g of seeds	23.4 ± 2.4 B	17.4 ± 2.0 A	57.9 ± 1.2 D	39.9 ± 1.7 C

Note

All data are expressed as the average values of three replicates ± standard deviation. Different letters indicate the existence of statistical differences between cultivars (*p* < .05).

Abbreviation: TPI, Total polyphenol index.

The highest values of the total polyphenol index and the tannin concentration were obtained by Merlot followed in decreasing order by Cabernet Sauvignon, Garnacha, and Tempranillo. Merlot and Cabernet Sauvignon seeds released much more tannins than Garnacha or Tempranillo seeds. Specifically, Merlot seeds released around five times more and Cabernet Sauvignon released around 3.5 times more than the other two seed varieties.

In general, the proanthocyanidin concentration showed a similar trend as the tannin concentration, although the values were lower. These differences are probably related to the different method used for each analysis. Tannins were determined using the methylcellulose precipitation method (Sarneckis et al., 2006) whereas proanthocyanidins were measured by acid depolymerization in the presence of an excess of phloroglucinol (Kennedy & Jones, 2001). This last analytical method cannot cleave all the interflavanic bonds of the proanthocyanidins and therefore may underestim ate the real concentration (Foo, Lu, Howell, & Vorsa, 2000) No significant differences were found in the mean degree of polymerization (mDP) of the proanthocyanidins between the different varieties. However, some differences were observed in their galloylation percentages (%GAL). Specifically, the galloylation percentage was higher for the proanthocyanidins extracted from Tempranillo and Merlot seeds. In contrast, the lowest galloylation percentage was observed in Cabernet Sauvignon seeds, while Garnacha seed proanthocyanidins showed an intermediate galloylation level.

According to these data, it seems that the extraction of phenolic compounds, tannins, and proanthocyanidins from seeds was mainly influenced by the percentage of seed weight respect to berry weight (%SB) of each variety, since Merlot and Cabernet Sauvignon grapes, which have a higher SB%, released more of these compounds than the other two varieties. However, Merlot and Cabernet Sauvignon grape berries showed statistically similar %SB but Merlot seeds released higher amounts of tannins and proanthocyanidins. This indicates that Merlot seeds can release higher amounts of these phenolic compounds than Cabernet Sauvignon. In contrast, Garnacha and Tempranillo seeds released similar amounts of these compounds, although Tempranillo had a significantly greater %SB. These data suggest that Garnacha seeds can release a higher amount of tannins than Tempranillo seeds by weight unit.

These different tannin releasing capacities of the seeds of the different varieties can be observed in Table 4, which shows the tannin concentration in relation to the seed weight. Specifically, Merlot seeds had the highest tannin releasing capacity followed, in decreasing order by Cabernet Sauvignon, Garnacha and Tempranillo seeds. It seems, therefore, that the tannin (and proanthocyanidin) concentration, which can be released from the seeds of the different cultivars, depends not only on the percentage of seed weight with respect to berry weight but also on the genetic characteristics of each cultivar, which probably condition the phenolic compound content of their seeds. In addition, the lignification level of the seeds, which is related to the grape maturity level, also affects the extractability of these phenolic compounds (Cadot, Minana‐Castello, & Chevalier, 2006; Gil et al., 2012).

Table 4 also shows the Astringency Index of the model wine solutions obtained by maceration of the seeds of each variety. Merlot seed macerated solution had the highest Astringency Index followed, in decreasing order, by Cabernet Sauvignon, Tempranillo, and Garnacha macerated solutions. In general, it seems that the astringency index of solutions was in line with the amount of phenolic compounds and tannin released. However, the Astringency Index of the Tempranillo seed macerated solution was significantly higher than that of the Garnacha seed solution, although its tannin and proanthocyanidin concentration were statistically similar. This could be due to a higher galloylation percentage of the proanthocyanidins released from Tempranillo seeds. When the Astringency Index was normalized by the seed weight of each variety, this parameter showed a similar trend as the tannin concentration normalized by gram of seeds. These data suggest that the percentage of seed weight with respect to the berry weight was the main determinant of the final tannin concentration and astringency of the solutions. However, the galloylation percentage also seems to contribute to the Astringency Index.

Table 5 shows the TPI and tannin concentration of the different wines elaborated for each variety: Grape Juice Wine (GJW), Seed Wine (SW), and Grape Wine (GW). To obtain the theoretical value of TPI released by seeds, the values corresponding to GJW were subtracted from those of SW in order to eliminate the influence of the phenolic compounds present in the grape juice after pressing. The same strategy was performed for tannins to determine the tannins released per seed under the real media conditions of each cultivar (pH, ethanol content, and titratable acidity characteristic of each cultivar) and under real alcoholic fermentation conditions. Evidently, these data should be considered with some precaution since they correspond to a theoretical calculation.

**TABLE 5:** Total polyphenol index, tannin concentration, and astringency index of the wines

Parameter	Garnacha	Tempranillo	Merlot	Cabernet Sauvignon
TPI				
GJW	11.7 ± 0.1 A	13.0 ± 0.2 B	10.9 ± 0.5 A	10.9 ± 0.8 A
SW	23.9 ± 0.3 A	25.5 ± 1.2 B	59.0 ± 1.5 D	43.9 ± 2.5 C
Theoretically released by seeds	12.2 ± 0.3 A	12.50 ± 1.2 A	48.1 ± 1.5 C	33.0 ± 2.5 B
GW	48.6 ± 0.4 A	63.8 ± 2.3 B	96.5 ± 0.9 D	90.4 ± 0.4 C
Tannins (mg/L)				
GJW	99 ± 23 A	419 ± 40 C	140 ± 28 AB	168 ± 13 B
SW	840 ± 91 A	1,085 ± 127 B	3,241 ± 122 D	1835 ± 82 C
Theoretically released by seeds	741 ± 91 A	667 ± 110 A	3,101 ± 122 C	1667 ± 48 B
GW	1979 ± 163 A	2,605 ± 100 B	4,057 ± 129 D	3,479 ± 147 C
Astringency index (mg/L Tannic Acid)				
GJW	80 ± 20 A	130 ± 30 A	80 ± 20 A	70 ± 30 A
SW	220 ± 20 A	280 ± 20 B	590 ± 20 D	510 ± 40 C
Theoretically released by seeds	140 ± 10 A	150 ± 20 A	510 ± 20 C	440 ± 30 B
GW	480 ± 10 A	510 ± 20 A	640 ± 20 B	660 ± 20 B

Note

All data are expressed as the average values of three replicates ± standard deviation. Different letters indicate the existence of statistical differences between cultivars (*p* < .05).

Abbreviations: GJW, Grape juice wine; GW, Grape wine; SW, Seeds wine; TPI, Total polyphenol index.

Considering these theoretical values, Merlot seeds released the highest TPI and tannin concentration followed in decreasing order by Cabernet Sauvignon, Tempranillo, and Garnacha seeds, although these last two varieties released similar amounts of tannins. In general, the theoretical concentration of tannin released by seeds in fermentation conditions showed a similar trend as that obtained in the model wine solution (Table 4) for all the varieties, although the values were lower. This was probably due to the presence of ethanol in the media. In the model wine solutions, alcoholic fermentation was not carried out and the ethanol content was high (13.5% v/v) during the entire maceration time. In contrast, SWs were obtained by real alcoholic fermentation and consequently the high ethanol content was only reached in the last steps of the fermentation. In addition, the ethanal produced by yeasts during alcoholic fermentation could also have originated the polymerization of proanthocyanidins causing the precipitation of the higher molecular weight polymers (Es‐Safi, Fulcrand, Cheynier, & Moutounet, 1999; Fulcrand, Doco, Es‐Safi, Cheynier, & Moutounet, 1996). According to these data, it seems that the amount of tannin released from seeds was mainly determined by the %SB, as in the model wine macerations.

In the case of grape wines (GW), the TPI and the tannin concentration were also higher for Merlot wine followed in decreasing order by Cabernet Sauvignon, Tempranillo, and Garnacha wines. It seems that the varieties that released more phenolic compounds and tannin from seeds, also released more tannin and phenolic compounds when skins and seeds were present in conditions of real winemaking. This fact is specially related with the %SB and probably also related with the berry size which can determine the skin proportion, as it has been reported by (Gil et al., 2015). However, the berry skin thickness of each cultivar can also determine the skin‐to‐flesh ratio (Gil et al., 2020).

Table 5 also shows the Astringency Index of the different wines of the different varieties. As it was proposed above for the TPI and tannin concentration, the Astringency Index of GJW was subtracted from that of the SW to determine the theoretical Astringency Index values of the substances released by seeds. In general, the astringency index obtained for the seed wines showed a similar trend as that obtained for the model wine solutions (Table 4); however, the index was lower, probably because the amount of tannin released was also lower. Consequently, the highest Astringency Index was again obtained for Merlot seeds followed in decreasing order by Cabernet Sauvignon, Tempranillo, and Garnacha seeds. The Astringency Index of the model wine solution macerations of Tempranillo seeds was higher than that of Garnacha seeds, whereas for the wines the theoretical astringency index of seeds for both cultivars was similar. These differences are probably because factors other than the phenolic compounds also play a role in the astringency. For example, the polysaccharide concentration (De Freitas, 2003; Watrelot, Schulz, & Kennedy, 2017), ethanol content (Fontoin, Saucier, Teissedre, & Glories, 2008) and pH (Obreque‐Slier, Pena‐Neira, & López‐Solis, 2012), which are very low in Garnacha wines, have been reported to be key factors for modulating wine astringency. In any case, the theoretical astringency index of seeds seems to be mainly determined by the amount of tannin released by seeds, and the %SB is probably the main determinant of the astringency of the wines of the different varieties. Nevertheless, other media conditions, such as pH, ethanol and polysaccharide content, may also affect wine astringency.

The Astringency Indices of Merlot and Cabernet Sauvignon wines in conditions of real winemaking (GWs) were similar and significantly higher than those of Tempranillo and Garnacha wines. As expected, the astringency of all GWs was higher than the astringency of SWs because skins also contribute to the tannin release. However, the increase in astringency of Merlot and Cabernet Sauvignon wines due to the presence of skins was lower than in the Tempranillo and Garnacha wines. This minor increase in astringency in Merlot and Cabernet Sauvignon GWs could be associated to a slightly higher maturity of these grapes that could favor the extraction of other compounds that can decrease the astringency perception, such as polysaccharides (Foo et al., 2000; Harbertson & Harwood, 2009).

Table 6 shows the proanthocyanidin concentration of the different wines elaborated with the four varieties. Once again the proanthocyanidin concentration of the GJW was subtracted from that of the SW to calculate the theoretical concentration of proanthocyanidins released by seeds. Similar calculations were performed to determine their theoretical values of mDP and %GAL considering the individual chromatograms. In general, the theoretical concentration of proanthocyanidin released by seeds showed a similar trend to the tannin concentration (Table 5). Specifically, the highest theoretical proanthocyanidin concentration value was obtained for Merlot followed in decreasing order by Cabernet Sauvignon, Garnacha, and Tempranillo, and the last two varieties had statistically similar values.

**TABLE 6:** Proanthocyanidins and related parameters of the wines

Parameter	Garnacha	Tempranillo	Merlot	Cabernet Sauvignon
Proanthocyanidins (mg/L)				
GJW	69 ± 1 A	285 ± 3 D	115 ± 3 C	73 ± 1 B
SW	297 ± 5 A	498 ± 7 B	1,204 ± 23 D	837 ± 47 C
Theoretically released by seeds	265 ± 8 A	258 ± 7 A	1,139 ± 24 C	803 ± 49 B
GW	920 ± 105 A	921 ± 83 A	1,393 ± 64 B	1,600 ± 39 C
mDP				
GJW	5.4 ± 0.1 C	12.3 ± 0.1 D	3.4 ± 0.2 A	5.0 ± 0.1 B
SW	3.7 ± 0.2 A	5.0 ± 0.3 B	4.0 ± 0.3 A	4.7 ± 0.1 B
Theoretically released by seeds	3.6 ± 0.2 B	3.0 ± 0.2 A	3.9 ± 0.3 B	4.6 ± 0.1 C
GW	6.8 ± 0.1 B	7.1 ± 0.1 C	5.8 ± 0.7 A	7.4 ± 0.2 C
% GAL				
GJW	18.5 ± 0.2 D	5.8 ± 0.1 A	12.6 ± 1.0 B	19.4 ± 0.5 C
SW	19.0 ± 0.4 D	10.8 ± 0.4 A	16.5 ± 0.3 C	13.0 ± 0.1 B
Theoretically released by seeds	20.6 ± 0.7 D	18.6 ± 0.6 C	17.3 ± 0.3 B	13.4 ± 0.3 A
GW	10.2 ± 0.6 C	6.0 ± 0.3 A	9.7 ± 0.6 BC	8.6 ± 0.7 B
% Prodelphinidins				
GJW	n.d	n.d	n.d	n.d
SW	n.d	n.d	n.d	n.d
Theoretically released by seeds	n.d	n.d	n.d	n.d
GW	22.3 ± 0.5 B	16.5 ± 0.5 A	22.6 ± 1.1 B	25.53 ± 0.71 C

Note

All data are expressed as the average values of three replicates ± standard deviation. Different letters indicate the existence of statistical differences between cultivars (*p* < .05).

Abbreviations: %GAL, percentage of galloylation; GJW, Grape juice wine; GW, Grape wine; mDP, mean degree of polymerization; SW, Seeds wine.

Some significant differences were observed in the theoretical values of mDP and %GAL of the proanthocyanidins from the seeds of the different varieties. The proanthocyanidins released by Tempranillo seeds had the lowest mDP followed in increasing order by Garnacha, Merlot and Cabernet Sauvignon seeds. Moreover, the %GAL was also different for the proanthocyanidins extracted from each cultivar. The highest %GAL was obtained for the proanthocyanidins extracted from Garnacha seeds followed in decreasing order by those of Tempranillo, Merlot, and Cabernet Sauvignon. These mDP and %GAL values differ somewhat from those reported for the seeds macerated in the model wine solution. This is probably because the different compositions of the grape juices (pH, ethanol content, titrable acidity, among others) may affect the proanthocyanidin extraction and also their structural changes. It has been described that higher ethanol content causes higher extraction of the proanthocyanidins with higher mDP (Henandez‐Jimenez, Kennedy, Bautista‐Ortín, & Gomez‐Plaza, 2012). It has also been reported that at a very acidic pH, the interflavan bonds are expected to rupture more easily due to the nucleophilic character of the molecules (Dallas, Hipólito‐Reis, Ricardo‐da‐Silva, & Laureano, 2003).

In general, the proanthocyanidin concentration of the GWs of the four cultivars showed a similar trend as that observed for the tannin concentration, although this time the highest value was obtained for Cabernet Sauvignon followed in decreasing order by Merlot, Tempranillo, and Garnacha, with the last two varieties having statistically similar values. As expected, the mDP of the proanthocyanidins of GWs was significant higher and the %GAL significantly lower than in the SWs. These differences are because skin proanthocyanidins have higher mDP and lower %GAL than seed proanthocyanidins (Prieur et al., 1994; Souquet et al., 1996). Table 6 also shows the %PD of the grape wines. The highest %PD was obtained in Cabernet Sauvignon followed in decreasing order by Merlot, Garnacha, and Tempranillo.

Table 7 shows the anthocyanin concentration of the different GWs measured by spectrophotometry. The wines elaborated with Merlot grapes showed the highest anthocyanin concentration followed in decreasing order by Cabernet Sauvignon, Tempranillo, and Garnacha wines. This table also shows the anthocyanin concentration analyzed by HPLC.

**TABLE 7:** Anthocyanin concentration and color parameters of grape wines

Parameter	Garnacha	Tempranillo	Merlot	Cabernet Sauvignon
Anthocyanins	110 ± 15 A	461 ± 31 B	762 ± 6 D	532 ± 28 C
HPLC				
Total anthocyanins	35 ± 7 A	121 ± 40 B	285 ± 14 D	177 ± 2 C
Anthocyanidins‐3‐*O*‐ monoglucosides	33 ± 7 A	108 ± 38 B	239 ± 8 C	120 ± 10 B
Accetylated anthocyanins	0.3 ± 0.1 A	1.5 ± 0.5 B	43.0 ± 2.0 C	52.0 ± 9.0 C
Cumarylated anthocyanins	1.7 ± 0.4 A	10.5 ± 5.1 B	20.0 ± 2.0 C	5.0 ± 1.0 B
Piranoanthocyanins	6.0 ± 3.0 A	13.4 ± 2.8 B	17.0 ± 3.0 B	32.0 ± 4.0 C
Color intensity	9.1 ± 0.1 A	18.1 ± 0.5 B	25.1 ± 0.7 C	25.0 ± 1.2 C
CIEL*a*b* Coordinates				
L*	55.0 ± 0.4 D	32.5 ± 0.2 C	29.2 ± 0.6 B	25.8 ± 1.5 A
C*	49.2 ± 0.4 A	57.2 ± 1.1 B	63.8 ± 0.1 C	58.6 ± 0.9 B
H*	3.2 ± 0.5 A	9.5 ± 0.5 B	22.0 ± 0.7 C	22.4 ± 0.3 C

Note

All data are expressed as the average values of three replicates ± standard deviation. Anthocyanin and pyranoanthocyanin concentrations are expressed in mg/L. Different letters indicate the existence of statistical differences between cultivars (*p* < .05). L*: Lightness; C*: Chroma; H*: Hue.

As a general rule, anthocyanidin‐3‐*O*‐monoglucosides and the total anthocyanin concentrations determined by HPLC‐DAD showed a similar trend as that measured by spectrophotometry, although the concentrations were lower. This is logical because the spectrophotometric analysis includes the contribution from other pigments in the measurement and therefore overestimates the total anthocyanin concentration, whereas the HPLC‐DAD methods only detect free anthocyanins (Rivas‐Gonzalo, Gutierrez, Hebrero, & Santos‐Buelga, 1992).

It stands out that Cabernet Sauvignon and Merlot wines have a higher concentration of acetylated anthocyanins than coumarylated anthocyanins whereas Garnacha and Tempranillo wines have higher concentration of coumarylated anthocyanins than acetylated anthocyanins. These differences in the proportion of acetylated and coumarylated anthocyanins have been described previously by other authors for Cabernet Sauvignon and Tempranillo wines (Gil et al., 2012; Otteneder, Holbach, Marx, & Zimmer, 2001). Moreover, the pyranoanthocyanin concentration was highest for Cabernet Sauvignon wines, followed in decreasing order by Merlot, Tempranillo, and Garnacha wines.

Table 7 also shows the color intensity and the CIEL*a*b* coordinates L*, C* and H*. In general, the color intensity and C* were in accordance with the anthocyanin concentration of the different wines. The only exception was the Cabernet Sauvignon wine, which had a similar color intensity as the Merlot wine although its anthocyanin concentration was significantly lower. This could be related to the higher concentration of pyranoanthocyanins observed in Cabernet Sauvignon wine and also to the copigmentation phenomena. As expected, L* showed the opposite trend to color intensity and C* although some minor differences were observed. The greater differences were found in H*. Specifically, Garnacha wines had the lowest H* value followed in increasing order by Tempranillo, Merlot, and Cabernet Sauvignon, and these last two values were statistically similar. In general, this behavior of H* can be related to the pH and pyranoanthocyanin concentration of the different wines since the lower the pH and the lower the pyranoanhocyanin concentration the lower the yellowish nuances (Brouillard & Dubois, 1997; De Freitas & Mateus, 2011). Moreover, the copigmentation phenomena can also produce a decrease in H* (Brouillard & Dangles, 1994; Gombau et al., 2019).

Merlot and Cabernet Sauvignon, which have the smallest berry size (100BW and 100 BV), resulted in wines with the deepest color and the richest anthocyanin concentration, whereas Garnacha and Tempranillo which have the largest berry size, resulted in the opposite. It seems, therefore, that the berry size is related to the anthocyanin concentration and the color intensity, as it have been described by Gil et al. (2015), although this relation is not lineal suggesting that skin thickness also could determine the extractability of these compounds from skins (Gil et al., 2020).

**FIGURE 2:**
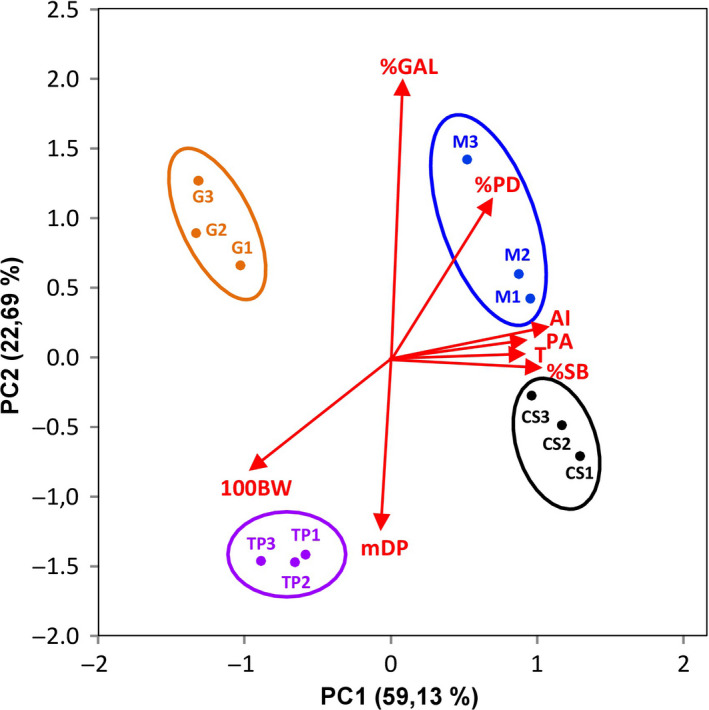
Plot of varimax‐rotated principal components analysis for the different grape wines. Abbreviations: %GAL, percentage of galloylation; %PD, percentage of prodelphinidins; %SB, percentage of seed weight with respect to berry weight; 100BV, volume of 100 berries; AI, Astringency Index; CS, Cabernet Sauvignon; G, Garnacha; M, merlot; mDP, mean degree of polymerization; PA, Proanthocyanidins; T, Tannins; TP, Tempranillo

A principal component analysis was performed to better understand which factors are the main determinants of the astringency of the grape wines (GW). Figure 2 shows the plot of varimax‐rotated principal components analysis of the different grape wines. This statistical analysis was performed with the parameters Astringency Index, tannins, proanthocyanidins, mDP, %GAL, %PD, %SB, and 100BW. Tannins and proanthocyanidins were included because they correlate very well with the Astringency Index. The mDP, %GAL, and %PD were included because they have been reported to be key factors in determining the astringency of proanthocyanidins (Sun et al., 2013; Vidal et al., 2003). Finally, %SB and 100BW were included to study the influence of the grape morphology. Other parameters were not considered in order to simplify the conclusions of the PCA. The first component explains 59.13% of the variance, and the second explains 22.69%, and therefore, the aggregate variance explained by the first two components was 81.82%.

The PCA enabled us to separate the different varieties. PC1 placed Merlot and Cabernet Sauvignon wines on the right and Garnacha and Tempranillo on the left. In contrast, PC2 placed Merlot and Garnacha wines at the top of the graph, whereas Cabernet Sauvignon and Tempranillo wines were placed at the bottom.

The loadings are shown as arrows, the length and direction of which indicate the contribution made by the two components. The arrow corresponding to the Astringency Index is directed toward the right, indicating that the samples placed further toward the right have higher astringency. As expected, the arrows corresponding to proanthocyanidins and tannins are also directed to the right, nearly overlapping with the astringency index arrow, confirming that these compounds are the main determinants of astringency perception. The %SB arrow is also directed to the right, in a very similar way to the arrows for the astringency index, tannins, and proanthocyanidins. Hence, all these parameters mainly contribute to PC1 and it seems that they are closely and positively correlated. These data indicate that the percentage of seed weight with respect to berry weight is determinant of the wine's final tannin concentration and consequently also of its astringency. The mDP and the %GAL arrows are directed downwards and upwards respectively, contributing mainly to PC2, with angles close to 90° with respect to the Astringency Index arrow. These data are somewhat surprising since both parameters have been described as factors that condition the astringency of the proanthocyanidins (Sun et al., 2013; Vidal et al., 2003). These data seem to indicate that wine astringency is more conditioned by the proanthocyanidin concentration than by the %GAL or mDP. The %PD arrow is directed toward the upper right of the graph and has a contribution from both axes. In contrast, the 100BW arrow is directed to the lower left of the graph in the opposite direction to the Astringency Index arrow. These data confirm that there is a negative correlation between the berry size and the tannin concentration of the wines (Gil et al., 2015) and consequently with their astringency. It seems that astringency index correlates better with %SB than with the berry weight.

## 4 CONCLUSION

It can be concluded that the morphology of the berry and more specifically the seed weight percentage with respect to the berry weight and the berry weight, had a clear effect on the chemical composition of the wines and consequently on their astringency perception. Therefore, Merlot and Cabernet Sauvignon wines, which showed the highest percentage of seed weight with respect to berry weight and smallest berry size (100 BW and 100 BV), were the wines with highest tannin concentration and consequently they had higher astringency. In contrast, Garnacha, and Tempranillo wines, which showed a lower percentage of seed weight with respect to berry weight and larger berry size, were the wines with the lowest tannin, and consequently they had lower astringency. It is evident that the genetic characteristics of each variety as well as environmental factors, viticulture practices and oenological procedures also affect the tannin concentration and astringency. Nevertheless, according to our results the seed weight percentage with respect to the berry weight (%SB) seems to be one of the main determinants of the final tannin concentration and astringency of red wines.

## CONFLICT OF INTEREST

The authors declare no conflict of interest.

## ETHICAL STATEMENT

This study does not involve any human or animal testing.
